# Supplementary material for: Terabyte-scale supervised 3D training and benchmarking dataset of the mouse kidney
Source: Sci Data. 2023 Aug 3;10:510. doi: 10.1038/s41597-023-02407-5 (PMC10400611; doi:10.1038/s41597-023-02407-5)
Supplement: Supplementary file 1 — Supplementary Information [file 41597_2023_2407_MOESM1_ESM.docx]

Supplementary Information

**Terabyte-scale supervised 3D training and benchmarking dataset of the mouse kidney**

**Authors**

Willy Kuo^1,2,*^, Diego Rossinelli^1,2,*^, Georg Schulz^3^, Roland H. Wenger^1,2^, Simone Hieber^3^, Bert Müller^3,†^, Vartan Kurtcuoglu^1,2,†^

**Affiliations**

1. Institute of Physiology, University of Zurich, Zurich, Switzerland

2. National Centre of Competence in Research, Kidney.CH, Zurich, Switzerland

3. Biomaterials Science Center, Department of Biomedical Engineering, University of Basel, Allschwil, Switzerland

* These authors contributed equally to this work

† These authors jointly supervised this work

Corresponding author: Vartan Kurtcuoglu (vartan.kurtcuoglu@uzh.ch)

**Table of Contents**

[Materials and Suppliers List 2](#_Toc139984849)

[Detailed Methods 4](#_Toc139984850)

[References 12](#_Toc139984851)

## Materials and Suppliers List

#### **Perfusion reagents**

| Phosphate Buffered Saline (PBS) | Oxoid Phosphate Buffered Saline Tablets (Dulbecco A)  BR0014G, ThermoFisher Scientific, United States |
| --- | --- |
| Ketamine 100 mg/ml | Ketasol®-100 ad us. vet., injection solution  Dr. E. Graeub AG, Switzerland |
| Xylazine 20 mg/ml | Xylazin Streuli ad us. vet., injection solution  Streuli Pharma AG, Switzerland |
| Paraformaldehyde | Paraformaldehyde prilled, 95%  441244, Sigma Aldrich, Germany |
| Mineral oil | Mineral oil, light oil (neat), BioReagent  M8410, Sigma Aldrich, Germany |

#### **Surgery Tools**

| Fine scissors | Vannas Spring Scissors - 2.5mm Blades  15000-08, Fine Science Tools, Germany |
| --- | --- |
| Arterial clamp | Micro Serrefine - 10 × 2 mm  18055-01, Fine Science Tools, Germany |
| Arterial clamp applying forceps | Micro-Serrefine Clip Applying Forceps  18057-14, Fine Science Tools, Germany |
| Vessel dilating forceps | S&T Vessel Dilating Forceps - 11cm  00125-11, Fine Science Tools, Germany |
| Angled forceps | S&T 0.3mm × 0.25mm Forceps  00649-11, Fine Science Tools, Germany |
| Straight forceps | Rubis Switzerland Tweezers 5-SA  232-1221, VWR, United States |
| Silk suture for ligations | Non-Sterile Silk Suture Thread 5/0  18020-50, Fine Science Tools, Germany |

#### **Perfusion Consumables**

| 1 ml syringe | Injekt F 1 ml  9166017V, B. Braun, Germany |
| --- | --- |
| 26 G needle | Sterican 26 G × ½ “  466 5457, B. Braun, Germany |
| 10 ml syringe Luer Lock | NORM-JECT 10 ml (12 ml)  4100-X00V0, Henke Sass Wolf, Germany |
| 50 ml syringe Luer Lock | Omnifix 50 ml (60 ml)  4617509F , B. Braun, Germany |
| 1.2 µm syringe filter | Chromafil Xtra PET-120/25  729229, Macherey-Nagel, Germany |
| 3-way stopcock | Discofix C 3-way Stopcock  16494C, B. Braun, Germany |
| 21 G butterfly needle | Venofix Safety G21  4056521-01, B. Braun, Germany |
| 0.5 ml centrifugation tubes | PCR Single tubes, PP, 0,5 ml  781310, Brand, Germany |
| 1.5 ml centrifugation tubes | Micro tube 1.5ml  72.690.001, Sarstedt, Germany |

#### **Vascular casting**

| PU4ii | PU4ii VasQtec, Switzerland |
| --- | --- |
| 1,3-Diiodobenzene | 1,3-Diiodobenzene  475262, Sigma Aldrich, Germany |
| 2-Butanone | 2-Butanone 360473, Sigma Aldrich, Germany |

## Detailed Methods

#### Optimized vascular casting technique

Standard transcardial perfusions as used for whole animal vascular casting are unable to fill the renal vascular tree in its entirety, as most of the resin will flow to the outlet via lower resistance paths, such as the liver. As a result, resin droplets enclosed within residual water can be observed in venous vessels (Supplemental Figure S1).


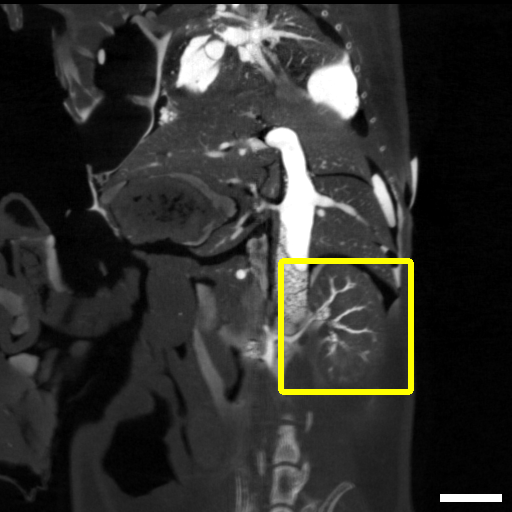

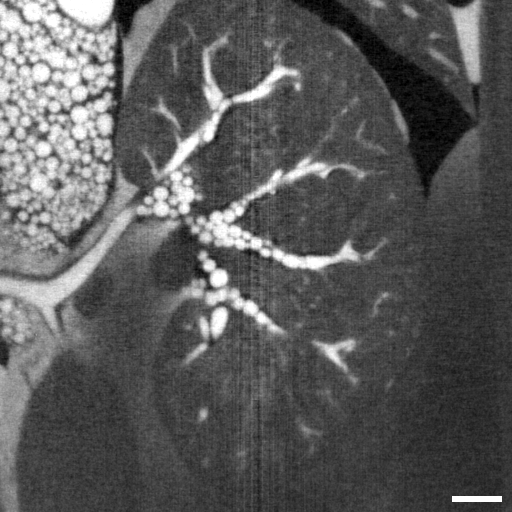

*Supplemental Figure S1: Results of an unoptimized transcardial vascular casting. Resin droplets enclosed in residual water can be observed in the renal venous vessel tree and the vena cava. A: Overview showing abdomen and thorax. Scale bar: 5 mm. B: Magnified view of the region of interest marked in yellow. Scale bar: 1 mm.*

**A**

**B**

Optimized perfusion techniques are required, where all resin flow is diverted to the organ of interest and lower resistance pathways are closed off via ligations.^1^ The herein presented method is derived from the isolated perfused kidney technique^2^ and was adapted for vascular casting.

#### Preparation

21 G butterfly needles were blunted by filing down their needle points with a metal file. Needles smaller than 21 G should not be employed for vascular casting, as they may not support the necessary flow rates. The outer surfaces of the needle were deburred with gentle filing in order to remove sharp edges or bumps, which may damage the vessel during needle insertion. The inside surfaces were deburred with a 26 G needle tip, in order to prevent flow restrictions.

The butterfly needle was then connected to a 3-way stopcock for flow control, which in turn was connected to 2.5 m long silicon tubing via Luer lock connector. A 50 ml syringe was connected to the silicone tubing to serve as a reservoir. The contraption was flushed with water until all air bubbles were removed, then filled with phosphate-buffered saline (PBS). The reservoir was hung at 2 m height above the working space to provide 150 mmHg of hydrostatic pressure.

2-Butanone should be degassed before use. Per mouse, 2.4 g of 1,3-diiodobenzene were dissolved in 7.5 g of 2-butanone and mixed with 7.5 g PU4ii resin within 50 ml centrifugation tubes in preparation. This corresponds to an iodine concentration of 100 mg iodine / g in the final vascular cast. PBS and formaldehyde solutions (PFA) were kept in a 37 °C water bath.

#### Preparing ligations for diverting flow

Mice were anaesthetized with 120 mg/kg ketamine and 24 mg/kg xylazine, with additional doses of 25 mg/kg ketamine and 4 mg/kg xylazine given after 15 min if surgical tolerance was not achieved. The animal was fixed to a foam board, which would allow fixation of the butterfly needle with pins at a later stage. The abdomen was opened with a vertical cut, and the intestine was moved to the right side (from the observer’s point of view). Throughout the entire surgery, the kidneys were wetted with 37° C PBS to prevent drying and premature coagulation. A part of the mesentery, visible as small transparent membrane, connects the intestine to the liver. This membrane was cut, so that the intestine could be moved entirely out of the way to the right without tearing the liver. A small needle cap was put under the back of the mouse to push up the region containing the superior mesenteric artery (SMA, Supplemental Figure S2). This vessel can be identified by the white tubing surrounding it, which is part of the mesentery, and should not be confused with venous vessels embedded in the surrounding fatty tissue. Fatty tissue does not form a distinct tubing and is more yellowish-white in color. The superior mesenteric artery can then be followed to find the proper ligation position at the abdominal aorta, which is anterior to the T-section where the superior mesenteric artery branches off of the abdominal aorta (AA, Supplemental Figure S2).

Fatty tissue around the ligation point was removed so that a 5/0 silk suture greased with mineral oil could be passed below the abdominal aorta. A constrictor knot ligation was prepared, but not closed (L1, Supplemental Figure S2). A second constrictor knot ligation was prepared at the mesenteric artery (L2, Supplemental Figure S2). The standard surgeon’s throw may not be used for these ligations, as it has been found to open at the pressures used in the vascular casting procedure. In an *in vitro* model, it has been found to leak at pressures as low as 33 mmHg, whereas the constrictor knot holds sufficiently tight at perfusion pressures of 363 mmHg under the same conditions.^3^

*Supplemental Figure S2: Schematic overview of the surgery steps necessary to perfuse kidneys (Ki). Constrictor knot ligations (L1, L2) around the abdominal aorta (AA) and superior mesenteric artery (SMA) were prepared. A third ligation (L3) was prepared posterior to the renal arteries (RA) and renal veins (RV). The abdominal aorta was clamped (Cl), and an incision was made to insert a blunted butterfly needle (In, BN), which was then fixed in place with the prepared ligation (L3). The vessel clamp (Cl) was removed, the remaining ligations (L1, L2) were closed and an incision was made into the vena cava (VC) to serve as an outlet (Out).*

#### Preparing ligation for needle insertion

The ideal needle insertion point (In, Supplemental Figure 2) in the abdominal aorta is posterior to the renal arteries (RA, Supplemental Figure 2). The longer the vessel segment between the insertion point and the renal arteries, the more securely the needle can be held in place by the ligation (L3, Supplemental Figure 2). In addition, more space allows additional attempts of inserting the needle to be taken further upstream the vessel if necessary. In practice, the needle ligation point will have to be chosen as a compromise based on where the abdominal aorta can be separated from the vena cava (VC, Supplemental Figure S2) for the ligation to be tied around the vessel, which is typically easier to perform closer to the renal arteries.

The vena cava can be readily identified as the largest vessel within the abdomen. Typically, it will be covered by fatty tissue, which should be split with tweezers to obtain access. The abdominal aorta can typically be found underneath the vena cava, towards the right side. Both of these blood vessels are surrounded by a fascia tubing, which needs to be cut or split with tweezers. The abdominal aorta can be grabbed gently with tweezers and pulled to the side without damaging the vessel. This may reveal a gap between the abdominal aorta and vena cava, where it’s possible to cut through the fascia tubing without injuring either vessel. Note that the vena cava may not be grabbed at any point, as it is very likely to burst. While leakage through the vena cava does not disturb the vascular casting process later on, as it will opened as an outlet anyways, the resulting bleeding will disturb the operator’s vision during the surgery until bleeding subsides, and lead to premature death of the animal.

If there is no gap large enough for surgical scissors to cut to allow separation of the two vessels, one should grab a part of the fascia tubing with one set of tweezers without grabbing either vessel, then grab onto the same part with a second set and gently pull the fascia tubing apart. Care needs to be taken to avoid exerting pulling force on the vena cava, as this may lead to vessel injury. This means that several small splits should be employed, rather than one big one. The gap created by this process may then be large enough to be cut through with surgical scissors, or may already be large enough on its own to pass through a greased silk suture.

A ligation was prepared around the abdominal aorta after the above process, but not closed yet (L3, Supplemental Figure S2). The abdominal aorta was then clamped between the ligation and the renal arteries to stop blood flow (Cl, Supplemental Figure S2). The vena cava may be partially clamped as well, in case that there is no full separation of the two vessels at this location.

#### Needle insertion and initial flushing

A small incision was made into the abdominal aorta with as much distance of the ligation as possible, to preserve as much vessel length as possible (In, Supplemental Figure S2). The incision should cut through 50 % of the vessel. Smaller cuts make it difficult to insert the vessel dilating forceps into the hole, larger cuts result in a more flaccid vessel that does not stay in place well during insertion.

Vessel dilating forceps were inserted into the hole, the vessel spread and the blunted 21 G butterfly needle (BN, Supplemental Figure S2) inserted between the forceps arms until it reached under the prepared ligation, and pushed against the vessel clamp. The butterfly needle was pinned behind its wings to prevent the needle to slip out of the vessel. The constrictor knot ligation (L3, Supplemental Figure S2) was then closed tight and the vessel clamp removed. The ligation of the superior mesenteric artery and ligation of the upper abdominal aorta were then closed as well.

A small window was cut into the vena cava some distance away from the renal veins to prevent them from collapsing under low pressure (Out, Supplemental Figure S2). For beginners, it is recommended to cut posterior to the ligation holding the needle in place (L3, Supplemental Figure S2), to avoid any consequences of accidentally cutting into the abdominal aorta. The kidneys were then flushed with 10 ml 37 °C PBS to remove the blood, then with 50 ml 37 °C 4 % formaldehyde in PBS (PFA) solution at 150 mmHg hydrostatic pressure. Flow rates can be observed based on the speed of drainage of the graduated syringe serving as reservoir, and typically achieve 5 ml / min during this step. This allows for a 10 min time window to prepare the PU4ii mixture, which can be extended by adding additional PFA solution to be perfused if required. Ideally, kidneys assume a pale coloration without visible blood patches after this step. (Supplemental Figure S3)


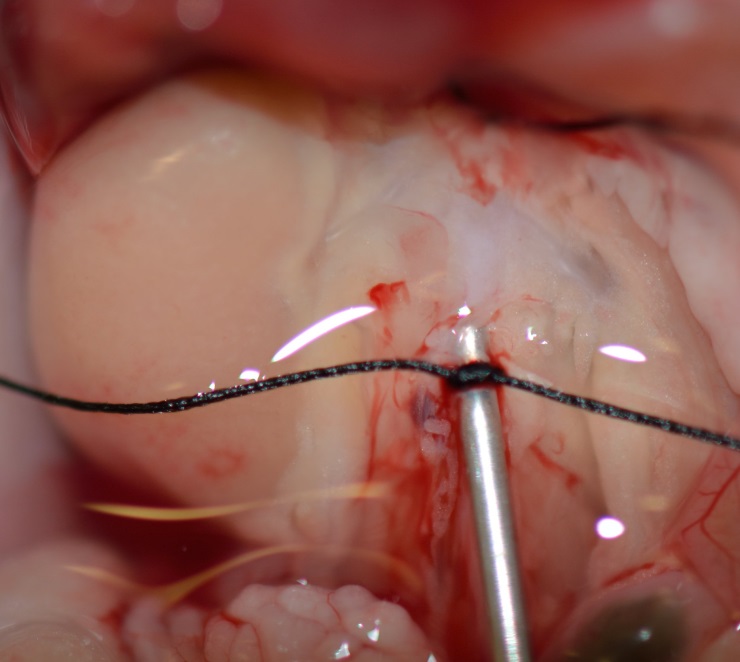


*Supplemental Figure S3: Right mouse kidney after perfusion with formaldehyde solution. Pale coloration of kidney and vena cava indicating successful flushing of blood (black and yellow arrows). One small blood patch can be observed on the bottom of the kidney (white arrow), which may indicate residual blood within the kidney, but may also be blood stuck to the outside of the organ as can be observed on top of the vena cava (yellow arrow).*

#### Vascular casting

1.3 g PU4ii hardener was added to the prepared mixture of 1,3-diiodobenzene, 2-butanone and PU4ii resin, and the mixture was filtered through a paper filter. The solution was degassed extensively in a vacuum chamber to minimize bubble formation during polymerization. It was then transferred to a 10 ml syringe with Luer lock. The 3-way stopcock was closed to stop flow, then the silicon tubing was disconnected and replaced with the resin-filled syringe.

The resin was perfused at a pressure of around 200 mmHg by actuating the syringe with a constant weight. The resin mixture was perfused until no more water bubbles could be observed exiting the outlet, which typically occurred after 3 ml of perfused volume. Typical flow rates are in the range of 0.2 ml / min during this step. If resin is observed exiting the abdominal aorta via the needle insertion point (In, Supplemental Figure S2), the ligation knot should be held tight with tweezers throughout the procedure (L3, Supplemental Figure S2). Pressure was kept throughout the entire procedure until the resin mixture solidified.

Supraphysiological perfusion pressures up to 200 mmHg cause distension of the blood vessel diameters, as well as mechanical compression of adjacent tubular luminae. Perfusion pressures above 200 mmHg may lead to bursting of the vessels and bleeding of vascular casting resin into the renal capsule, which results in distortion of the outer shape^4^. As such, perfusion pressure has to be chosen as a compromise between achieving reliable filling of the vasculature and staying close to physiological pressure ranges. It is, therefore, crucial to employ pressure-controlled pumps rather than standard syringe pumps with constant flow rates.

After polymerization, kidneys were excised and stored in 15 ml 4 % PFA. Kidneys were trimmed of fat as much as was possible without disturbing the outer surface of the kidney, as the radiopaque 1,3-diiodobenzene within the PU4ii mixture could diffuse into white adipose tissue. This issue can, in principle, be resolved by employing µAngiofil, another microparticle-free vascular casting reagent, which became available commercially after we concluded our experiments and which does not show this behavior.^4^ We have, however, found that the recommended degassing procedure was insufficient to prevent the formation of large gas bubbles in the resulting cast.^5^ Since viscosity and contrast are otherwise comparable to our mixture and µAngiofil features considerably higher materials cost (270 $ per mouse compared to 20 $), we did not repeat our experiments with the new vascular casting resin.

For scanning, kidneys were embedded in 2 % agar in PBS in 0.5 ml polypropylene centrifugation tubes with an inner diameter of 6 mm and an outer diameter of about 7.9 mm. The agar solution was degassed briefly in vacuum after heating to 100 °C, and left to cool down to approximately 40 °C before embedding the kidney. Care needs to be taken to avoid the introduction of any gas bubbles into the agar, as these will expand under the high radiation doses employed in this work’s synchrotron radiation-based hard X-ray phase-contrast microtomography (SRµCT) imaging, resulting in movement artifacts.

#### ESRF ID19 SRµCT measurements

Kidneys were scanned at the ID19 tomography beamline of the European Synchrotron Radiation Facility (ESRF, Grenoble, France) using pink beam with a mean photon energy of 19 keV. Radiographs were recorded at a sample-detector distance of 28 cm with a 100 µm Ce:LuAG scintillator, 4× magnification lens and a pco.edge 5.5 camera with a 2560 × 2160 pixel array and 6.5 µm wide pixels, resulting in an effective pixel size of 1.625 µm and a field of view of 4.16 mm in width and 3.51 mm in height.

To extend the field of view, radiographs were acquired with an asymmetric rotation axis scheme: by offsetting the rotation axis, a single radiograph of half of the width of sample could be stitched with its 180° rotated equivalent of the other half. For registration, an approximately 200 pixel broad overlap was kept, resulting in extended field of view of 8 mm.

Six height steps were recorded for each kidney, with half of the vertical field of view overlapping between each height step. This resulted in fully redundant acquisition of the kidneys with the exception of the halves of the top and bottom height steps, which was later employed for ring artifact removal.

5125 radiographs were recorded for each height step with 0.1 s exposure time, resulting in a scan time of 10 min per height step or one hour for a whole kidney. This allowed us to remain below the time threshold at which gas bubbles would start to form within the agar, which was around the 15 min mark. 100 flat-field images were taken before and after each height step for flat-field correction. Data size of the radiographs was 55 GB per height step, or 330 GB per kidney (**D1**).

Images were reconstructed using the beamline’s in-house PyHST2 software^6^, using a Paganin-filter^7^ with a low δ/β ratio of 50 to limit loss in resolution and appearance of gradients close to large vessels.^8^ Registration for stitching two radiographs for the full field of view was performed manually with 1 pixel accuracy. All other parameters were kept on default settings. Data size of the reconstructed datasets was 193 GB per height step, or 1158 GB per kidney (**D2**).

#### Ring artifact removal

Physical phenomena such as dust on the scintillator and highly absorbing particles in the beam path can result in a deviation from the expected response of the detector, producing ring artifacts at those locations. Outliers in intensity in the recorded flat fields were segmented to calculate radius and coordinates of the ring artifacts by noise reduction with 2D continuous curvelets, followed by thresholding. The redundant acquisition of the central four height steps allowed us to replace corrupted data with a weighted average during stitching. The grey values of the individual slices were zeroed in the presence of the rings, summed up and divided by the number of uncorrupted signals. In the outer slices, where no redundant data was available, and in locations where rings coincided in both height steps, we employed a discrete cosine transform-based (DCT) inpainting technique with a simple iterative approach, where we picked Gaussian smoothing kernels progressively smaller in size and reconstructed the signal in the target areas by smoothing the signal everywhere at each iteration. The smooth signal in the target areas is then combined with the original signal elsewhere, to form a new image. In the next iteration, in turn, the new image is then smoothed to rewrite the signal at the target regions. The final inpainted signal exhibits multiple scales, since different kernel widths are considered at different iterations, starting with closing the gaps with the largest frequencies and progressive refinements with higher frequencies.

The alignment for stitching the six stacks was determined by carrying out manual 3D registration and double checking against pairwise stack-stack phase-correlation analysis.^9^ The stitching process reduced the dataset dimension per kidney to 4608 × 4608 × 7168 pixels, totaling 567 GB (**D3**).

#### Continuous curvelets denoising

We performed image enhancement based on 3D discretized continuous curvelets^10^, in a similar fashion as Starck et al.^11^ but with second generation curvelets (i.e. no Radon transform) in 3D. The enhancement was carried out globally by leveraging the Fast Fourier Transform with MPI-FFTW^12^, considering about 100 curvelets. The “wedges” (curvelets in the spectrum) have a conical shape and cover the unit sphere in an approximately uniform fashion. For a given curvelet, a per-pixel coefficient is obtained by computing an inverse Fourier transform of its wedge and the image spectrum. We then truncated these coefficients in the image domain against a hard threshold, and forward-transformed it again into the Fourier space, modulated the curvelets with the truncated coefficients and superposed them. The end effect of our approach has shown to squeeze the pixel intensities into a substantially more limited range of values, thus helping to avoid over- and under-segmentation of large and small vessels, respectively. A threshold-based segmentation followed the image enhancement. The enhancement parameters and threshold were manually chosen by examining six randomly chosen regions of interest. Spurious islands were removed by 26-connected component analysis, and cavities were removed by 6-connected component analysis, yielding a fully connected blood vessel segment (**D5**). A different threshold was chosen to derive the tubule segment (**D6**). As the tubule segment contained discontinuities in regions where tubules were compressed by nearby distended large blood vessels, connected component analysis could not be applied.

#### Maximum inscribed ball

We extended the thickness measurement algorithm proposed by Hildebrand and Ruegsegger^13^, based on subpixel-accurate signed distance transformation, in order to take advantage of the aggregate compute power of data centers with Message Passing Interface (MPI).

#### Manual annotations for glomeruli and perirenal fat

To generate manual training data for glomeruli, three selected regions of interest (ROI) 512 × 256 × 256 voxels in size were segmented by manual contouring, using the Freehand Selection Tool in Fiji/ImageJ.^14^ The resulting binary masks were viewed and corrected in all three dimensions multiple times in order to minimize slice-by-slice discontinuities common to single-dimension contouring (“stacks of pancakes”). Residual discontinuities remain in the last manually contoured dimension, which could in principle be removed by smoothing, but were kept as is in order to keep the annotations fully manual (**D4**).

For removing perirenal fat from the blood vessel segment for visualization, three different regions of interest were annotated. Manual work was reduced by providing an automated initial guess of what was believed to be fat. For this, the image was strongly low-pass filtered and thresholded. The thickness transform of the resulting binary mask was calculated and thresholded to provide the initial guess, which was then corrected by manual annotation (**D9**).

#### Scattering transform

In order to detect glomeruli and remove perirenal fat from the blood vessel segment, we used a ML-based approach based on invariant scattering convolution networks.^15^ The scattering transform allowed us to design networks producing feature maps that are stable under small deformations. Although these networks are not steerable in the strictest sense, since they are not relying on irreducible representations of SO3, the produced features are equivariant under the action of a discrete subgroup of SO3 (regular representation).

The manually annotated regions of interest represent 0.06% of the image. The training data were supplemented by additional regions of interest which contained no glomeruli or no fat at all, and thus did not require manual annotation. The supervised set was then used to train a hybrid algorithm that relied on a 3D scattering transform convolutional network topped with a dense neural network. Gray values of the images were convolved with kernels with specific band pass properties and orientation, then contracted using complex modulus to generate intermediate scalar features. These features were further convolved with a low pass filter kernel to enforce the emergence of local invariances. The generated feature maps were fed to a dense, feed-forward neural network. The neural network processes each pixel independently to come up with a Boolean answer for each pixel. The neural network inference was run everywhere before further processing the foreground.

The scattering transform relied upon ad-hoc designed 3D kernels (Morlet’s wavelet with different sizes and orientations) that uniformly covered all directions at different scales, following the original work on the invariant scattering convolution networks.^15^ In the scattering convolutional network, filter nonlinearities were obtained by taking the magnitude of the filter responses and convolving them again with the kernels in a cascading fashion. These nonlinearities are designed to be robust against small Lipschitz-continuous deformations of the image.^15^

As opposed to our curvelet-based image enhancement approach, we decomposed the image into image cubic tiles, then applied a windowed -- thus local -- Fourier transform on a tile by considering a region about twice as large by width around the tile. While it would be possible to use a convolutional network based upon a global scattering transform, it would have incurred a very large amount of features that were to be consumed at once, leading to an intermediate footprint of about one Petabyte that would exceed the available RAM capacity of the cluster.

The scattering transform convolutional network produced a stack of a few hundreds of scalar feature maps, per pixel. We would like to note that if considered as a “fiber bundle”^16,17^, the feature map stack is equivariant under the symmetry group of rotations (i.e. the stack is a regular representation of SO(3)). This property can be exploited by further processing the feature maps with a dense neural network with increased parameter sharing across the hidden layers, making the output layer invariant to rotations.

The foreground representing the glomeruli was followed by a connected component analysis; the components are further examined by a tiny support vector machine to decide whether to retain or eliminate the connected component under examination (vector dimensionality of 8, training set of about 50 samples).^18^ The SVM feature vector included mass thickness percentiles (1, 10, 20, 40, 60, 80, 90, 99), fractal dimension and moment of inertia. Glomerular volume can be expected to lie within a restricted range.

#### Validation of glomeruli by domain expert

To reduce the workload to the level required to make validation of each glomerulus feasible, a volumetric visualization of the overlap between the blood vessel mask and the glomerular mask was generated for rapid evaluation of easy to recognize glomeruli or artefacts. In a first round, glomeruli were classified by their shape into three categories: 1. certain true positive with shape distortion, 2. certain true positive without shape distortion and 3. uncertain. Candidates of the third category, which constituted about 3 – 5 % of all machine-identified glomeruli, were then reviewed in a second round as an overlay over the original raw data on a slice-by-slice basis. These candidates were then assigned as false positives or as certain glomeruli of categories 1 or 2.

## References

1. Ghanavati, S., Yu, L. X., Lerch, J. P. & Sled, J. G. A perfusion procedure for imaging of the mouse cerebral vasculature by X-ray micro-CT. *Journal of Neuroscience Methods* **221**, 70–77 (2014).

2. Czogalla, J., Schweda, F. & Loffing, J. The Mouse Isolated Perfused Kidney Technique. *Journal of Visualized Experiments* 54712 (2016) doi:10.3791/54712.

3. Hazenfield, K. M. & Smeak, D. D. In vitro holding security of six friction knots used as a first throw in the creation of a vascular ligation. *Journal of the American Veterinary Medical Association* **245**, 571–577 (2014).

4. Hlushchuk, R. *et al.* Cutting-edge microangio-CT: new dimensions in vascular imaging and kidney morphometry. *American Journal of Physiology-Renal Physiology* **314**, F493–F499 (2018).

5. Kuo, W., Schulz, G., Müller, B. & Kurtcuoglu, V. Evaluation of metal nanoparticle- and plastic resin-based x-ray contrast agents for kidney capillary imaging. in *Developments in X-Ray Tomography XII* (eds. Müller, B. & Wang, G.) 23 (SPIE, 2019). doi:10.1117/12.2529414.

6. Mirone, A., Brun, E., Gouillart, E., Tafforeau, P. & Kieffer, J. The PyHST2 hybrid distributed code for high speed tomographic reconstruction with iterative reconstruction and a priori knowledge capabilities. *Nuclear Instruments and Methods in Physics Research Section B: Beam Interactions with Materials and Atoms* **324**, 41–48 (2014).

7. Paganin, D., Mayo, S. C., Gureyev, T. E., Miller, P. R. & Wilkins, S. W. Simultaneous phase and amplitude extraction from a single defocused image of a homogeneous object. *J Microsc* **206**, 33–40 (2002).

8. Rodgers, G. *et al.* Optimizing contrast and spatial resolution in hard x-ray tomography of medically relevant tissues. *Appl. Phys. Lett.* **116**, 023702 (2020).

9. Guizar-Sicairos, M., Thurman, S. T. & Fienup, J. R. Efficient subpixel image registration algorithms. *Opt. Lett.* **33**, 156 (2008).

10. Candès, E. J. & Donoho, D. L. Continuous curvelet transform. *Applied and Computational Harmonic Analysis* **19**, 198–222 (2005).

11. Starck, J.-L., Murtagh, F., Candes, E. J. & Donoho, D. L. Gray and color image contrast enhancement by the curvelet transform. *IEEE Trans. on Image Process.* **12**, 706–717 (2003).

12. Frigo, M. & Johnson, S. G. The Design and Implementation of FFTW3. *Proceedings of the IEEE* **93**, 216–231 (2005).

13. Hildebrand, T. & Rüegsegger, P. A new method for the model‐independent assessment of thickness in three‐dimensional images. *Journal of Microscopy* **185**, 67–75 (1997).

14. Schindelin, J. *et al.* Fiji: an open-source platform for biological-image analysis. *Nat Methods* **9**, 676–82 (2012).

15. Bruna, J. & Mallat, S. Invariant Scattering Convolution Networks. *IEEE Trans. Pattern Anal. Mach. Intell.* **35**, 1872–1886 (2013).

16. Cohen, T. S. & Welling, M. Steerable CNNs. *arXiv:1612.08498 [cs, stat]* (2016).

17. Weiler, M., Geiger, M., Welling, M., Boomsma, W. & Cohen, T. 3D Steerable CNNs: Learning Rotationally Equivariant Features in Volumetric Data. *arXiv:1807.02547 [cs, stat]* (2018).

18. Suykens, J. A. K. & Vandewalle, J. Least Squares Support Vector Machine Classifiers. *Neural Processing Letters* **9**, 293–300 (1999).
